# Cost‐effectiveness of precision diagnostic testing for precision medicine approaches against non‐small‐cell lung cancer: A systematic review

**DOI:** 10.1002/1878-0261.13038

**Published:** 2021-07-19

**Authors:** Raymond Henderson, Peter Keeling, Declan French, Dave Smart, Richard Sullivan, Mark Lawler

**Affiliations:** ^1^ Diaceutics PLC Belfast UK; ^2^ Queen’s Management School Queen’s University Belfast UK; ^3^ Institute of Cancer Policy King’s College London & King’s Health Partners Comprehensive Cancer Centre UK; ^4^ Patrick G. Johnston Centre for Cancer Research Queen’s University Belfast UK

**Keywords:** biomarker, cost‐effectiveness analysis, economic evaluation, non‐small‐cell lung cancer, precision diagnostic test, precision medicine

## Abstract

Precision diagnostic testing (PDT) employs appropriate biomarkers to identify cancer patients that may optimally respond to precision medicine (PM) approaches, such as treatments with targeted agents and immuno‐oncology drugs. To date, there are no published systematic appraisals evaluating the cost‐effectiveness of PDT in non‐small‐cell lung cancer (NSCLC). To address this gap, we conducted Preferred Reporting Items for Systematic Reviews and Meta‐Analyses searches for the years 2009–2019. Consolidated Health Economic Evaluation Reporting Standards were employed to screen, assess and extract data. Employing base costs, life years gained or quality‐adjusted life years, as well as willingness‐to‐pay (WTP) threshold for each country, net monetary benefit was calculated to determine cost‐effectiveness of each intervention. Thirty‐seven studies (50%) were included for analysis; a further 37 (50%) were excluded, having failed population‐, intervention‐, comparator‐, outcomes‐ and study‐design criteria. Within the 37 studies included, we defined 64 scenarios. Eleven scenarios compared PDT‐guided PM with non‐guided therapy [epidermal growth factor receptor (*EGFR*), *n* = 5; programmed death‐ligand 1 (PD‐L1), *n* = 6]. Twenty‐eight scenarios compared PDT‐guided PM with chemotherapy alone (anaplastic lymphoma kinase, *n* = 3; *EGFR*, *n* = 17; PD‐L1, *n* = 8). Twenty‐five scenarios compared PDT‐guided PM with chemotherapy alone, while varying the PDT approach. Thirty‐four scenarios (53%) were cost‐effective, 28 (44%) were not cost‐effective, and two were marginal, dependent on their country’s WTP threshold. When PDT‐guided therapy was compared with a therapy‐for‐all patients approach, all scenarios (100%) proved cost‐effective. Seven of 37 studies had been structured appropriately to assess PDT‐PM cost‐effectiveness. Within these seven studies, all evaluated scenarios were cost‐effective. However, 81% of studies had been poorly designed. Our systematic analysis implies that more robust health economic evaluation could help identify additional approaches towards PDT cost‐effectiveness, underpinning value‐based care and enhanced outcomes for patients with NSCLC.

## Graphical abstract

Precision diagnostic testing guides therapy to non‐small‐cell lung carcinoma (NSCLC) patients who will best respond, increasing both quantity and quality of life. Our analysis demonstrates the inherent value of precision medicines for NSCLC patients in over half of cases. However, to build the health economic evidence base further, cost‐effectiveness analyses should be appropriately designed to maximise the potential of precision diagnostic testing.

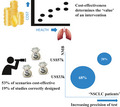

**Abbreviations**
*ALK*
: anaplastic lymphoma kinase
ARMS: amplification refractory mutation system
ASCO: American Society for Clinical Oncology
AUD$: Australian dollars
CAD$: Canadian dollars
CEA: cost‐effectiveness analysis
CET: cost‐effectiveness threshold
CHF: Swiss francs
CN: China
*EGFR*
: epidermal growth factor receptor
FISH: fluorescent in situ hybridization
HK$: Hong Kong dollars
HRM: high‐resolution melt
ICER: incremental cost‐effectiveness ratio
IHC: immunohistochemistry
IO: immuno‐oncology
ISPOR: International Society for Pharmacoeconomic Outcomes Research
LYG: life year gained
*MET*
: mesenchymal epithelial transition factor
MTS: multiplex targeted sequencing
NGS: next‐generation sequencing
NMB: net monetary benefit
NSCLC: non‐small‐cell lung carcinoma
OWSA: one‐way sensitivity analysis
PAP: patient access programme
PD‐L1: programmed death‐ligand 1
PDT: precision diagnostic test
PICOS: population, intervention, comparison, outcome, and study design
PM: precision medicine
PSA: probabilistic sensitivity analysis
QALY: quality‐adjusted life‐year
RCT: randomized controlled trial
*RET*
: rearranged during transfection
*ROS1*
: c‐ros oncogene 1
S$: Singapore dollars
T790M: EGFR gatekeeper mutation
TKI: tyrosine kinase inhibitor
WTP: willingness‐to‐pay

## 1 Introduction

The World Health Organization (WHO) lists lung cancer as the most common cancer and leading cause of cancer death (1.76 million globally) [1]. In the USA, lung cancer was projected to cause 140 730 cancer deaths in 2020 (almost a quarter of total cancer deaths), with projected lung cancer deaths in the EU at 182 600 in 2020 [2, 3]. Relative survival of lung cancer is poor, at 39% and 13% for 1‐ and 5‐year survival, respectively. Non–small‐cell lung cancer (NSCLC) accounts for 85% of lung cancer cases [4, 5].

Since 2009, several classes of drugs for NSCLC have been approved for use, all with accompanying precision diagnostic tests (PDT). These include tyrosine kinase inhibitors (TKI) against epidermal growth factor receptor (*EGFR*; gefitinib, erlotinib, afatinib, dacomitinib and osimertinib), TKI against anaplastic lymphoma kinase (*ALK*; crizotinib, ceritinib, alectinib, brigatinib, and lorlatinib), v‐raf murine sarcoma viral oncogene homolog B1 (*BRAF*; dabrafenib/trametinib), c‐ros oncogene 1 (*ROS1*; crizotinib or entrectinib), mesenchymal epithelial transition factor (*MET*; capmatinib or tepotinib), rearranged during transfection proto‐oncogene (*RET*; selpercatinib), neurotrophic tropomyosin receptor kinase (*NTRK*; entrectinib or larotrectinib) and immuno‐oncology (IO) drugs (pembrolizumab, nivolumab, atezolizumab and durvalumab). The American Society of Clinical Oncology (ASCO) and Ontario Health guidelines state that the 60% of stage IV NSCLC patients with actionable mutations (*EGFR*, *ALK*, *BRAF*, *ROS1*, *MET*, *RET* and *NTRK)* should be offered the corresponding precision medicine (PM) that targets these abnormalities, and the remaining 40% without driver mutations should be offered immunotherapy, dependent on programmed death‐ligand 1 (PD‐L1) tumour proportion score test results [6, 7]. However, the costs of these new agents are proving unsustainable, in both unregulated markets and in socialised healthcare systems [8, 9]. However, this challenge has to be set against improved patient outcomes observed using these new targeted agents [10]. Quantifying the impact requires a value assessment of a PM intervention to both the patient and the payer [11, 12]. This requires some form of health economic evaluation, as part of a health technology assessment process.

To understand the health economic evaluation landscape of PDT‐guided PM, we undertook a systematic review of the evidence available for value‐based policymaking in this domain. Our hypothesis is that PDT, while a fraction of the cost of their associated PM, provide substantial value in terms of health benefits.

## 2 Methods

The review is registered with PROSPERO (registration number: CRD42020171234) as per Preferred Reporting Items for Systematic Reviews and Meta‐analyses guidelines [13]. The methodology followed was similar to a previous paper by the authors [14].

### 2.1 Search strategy

Utilising the PICOS framework (population, intervention, comparator, outcome, study design), we formulated the research question: ‘What is the cost‐effectiveness of precision diagnostic testing (PDT) for guiding therapy in non‐small‐cell lung cancer?’ PICOS was employed to develop a search limited to studies that performed economic evaluation of patients diagnosed with NSCLC who were subsequently stratified for treatment selection based on a PDT result. The search was conducted for studies reported between 1 January 2009 and 31 December 2019. We searched MEDLINE, Embase, Cochrane Library, SCOPUS, Web of Science, NHS Economic Evaluation Database (EED) and Econlit. Meeting presentations were also searched for the same period in the ASCO and International Society for Pharmacoeconomics and Outcomes Research (ISPOR) websites (see Table 1).

**Table 1:** Search results from 10 databases and hand searching.

Search term	Database	Identified	Screened	Duplicates	Eligible
See MEDLINE	MEDLINE	4782	11		22
See EMBASE	Embase	13 698	38	5	5
Cost‐effectiveness analysis of testing for lung cancer therapy	PubMed	34	32		21
Cost‐effectiveness analysis of testing for lung cancer therapy	Cochrane	28	10	3	1
Cost‐effectiveness analysis + testing + lung cancer therapy	SCOPUS	33	32	9	5
Cost‐effectiveness analysis + testing + lung cancer therapy	Web of Science	33	18	8	2
Testing + lung cancer therapy	NHS EED	38	0	0	0
Cost‐effectiveness analysis + testing + lung cancer therapy	EconLit	1	1	0	0
Cost‐effectiveness analysis of testing for lung cancer therapy	ASCO	6	4	0	0
Cost‐effectiveness analysis of testing for lung cancer therapy	ISPOR	21	18	0	1
Systematic reviews of economic evaluations in PM or lung cancer	Hand searches	49	49	11	17
	Total:	18 723	213	36	74

### 2.2 Search terms for MEDLINE and Embase

MEDLINE and Embase (OvidSP): 2009 to 2019 week 52.

Searched 1 January 2020.

1. exp Lung Neoplasms/
2. ((lung or bronchial or nsclc) adj3 (adenocarcinoma$ or adenoma$ or cancer$ orcarcinoma$ or lesion$ or malignan$ or meta‐sta$ or metasta$ or neoplas$ or oncolog$ or sarcoma$ or tumo?r$)).ti,ab,ot,hw.
3. NSCLC.ti,ab,ot.
4. or/1–3
*Polymorphism, Genetic/ or *Mutation/ or *Genotype/
6. (EGFR or KRAS or ALK or PD‐L1 or MSI or TMB or PD‐1).mp. [mp=title, abstract, original title, name of substance word, subject heading word, floating sub‐heading word, keyword heading word, organism supplementary concept word, protocol supplementary concept word, rare disease supplementary concept word, unique identifier, synonyms]
7. (((HRM test or HRMA test or sanger sequencing or pyrosequencing or high resolution) adj3 melt) or next generation sequencing).mp. [mp=title, abstract, original title, name of substance word, subject heading word, floating sub‐heading word, keyword heading word, organism supplementary concept word, protocol supplementary concept word, rare disease supplementary concept word, unique identifier, synonyms]
8. (DNA profil* or Mutligene assay or expression profil* or DNA mutational analysis or genetic testing or germ‐line mutation or nucleotide sequence or genetic screening or germline mutation or ((germline or germ‐line) adj8 mutation)).mp. or ((genetic* adj test*) or (mutation* adj analysis)).tw. [mp=title, abstract, original title, name of substance word, subject heading word, floating sub‐heading word, keyword heading word, organism supplementary concept word, protocol supplementary concept word, rare disease supplementary concept word, unique identifier, synonyms]
9. (immunohistochemistry or ihc).mp. [mp=title, abstract, original title, name of substance word, subject heading word, floating sub‐heading word, keyword heading word, organism supplementary concept word, protocol supplementary concept word, rare disease supplementary concept word, unique identifier, synonyms]
10. biomarker.mp.
11. ((genom$ or precision or personali$ or stratif$ or individuali$ or target$ or P4) adj (medic$ or treatment or therap$)).ti,ab,ot,hw.
12. 5 or 6 or 7 or 8 or 9 or 10 or 11
13. (Immunotherapy or Atezolizumab or Tecentriq or Durvalumab or Imfinzi or Nivolumab or Opdivo or Pembrolizumab or Keytruda).mp.
14. (Anti‐EGFR therapy or EGFRi or Erlotinib or Tarceva or Afatinib or Gilotrif or Gefitinib or Iressa or Osimertinib or Tagrisso or Dacomitinib or Vizimpro).mp.
15. (Entrectinib or Rozlytrek or Crizotinib or Xalkori or Ceritinib or Zykadia or Alectinib or Alecensa or Brigatinib or Alunbrig or Lorlatinib or Lorbrena).mp.
16. (Trametinib or Mekinist or Dabrafenib or Tafinlar).mp.
17. (angiogenesis inhibitors or Bevacizumab or Avastin or Ramucirumab or Cyramza).mp.
18. (Chemotherapy or Cisplatin or Carboplatin or Paclitaxel or Taxol or Abraxane or docetaxel or Taxotere or Gemcitabine or Gemzar or Vinorelbine or Navelbine or Etoposide or VP‐16 or Pemetrexed or Alimta).mp.
19. surgery.mp. [mp=title, abstract, original title, name of substance word, subject heading word, floating sub‐heading word, keyword heading word, organism supplementary concept word, protocol supplementary concept word, rare disease supplementary concept word, unique identifier, synonyms]
20. Radiotherapy.mp.
21. or/13–20
22. Costs.mp. and Cost‐Analysis/
23. Cost‐Benefit Analysis/
24. exp Models, Economic/
25. Quality‐Adjusted Life Year*.mp.
26. Economic Evaluation/
27. Cost‐Effectiveness Analysis/
28. Cost Utility Analyses/
29. Statistical Model/
30. (econom* or pharmacoeconomic* or pharmaco‐economic*).ti.
31. (economic evaluation* or economic review*).tw.
32. (cost* adj2 (util* or effective* or benefit? or analy*)).tw.
33. (health adj2 utilit*).tw.
34. (euroqol or eq5d or eq‐5d or hui or hui1 or hui2 or hui3).mp.
35. ((utility* adj2 (valu* or measure*)) or (time adj2 trade) or (standard adj2 gamble)).mp.
36. ((cost* or economic*) adj2 model*).tw.
37. (sensitivity analys$ or "willingness to pay" or quality‐adjusted life year$ or quality adjusted life year$ or quality‐adjusted life expectanc$ or quality adjusted life expectanc$).ti,ab.
38. (economic$ or cost or costs or costly or costing or costed or price or prices or pricing or priced or discount or discounts or discounted or discounting or ration$ or expenditure or expenditures or budget$ or afford$ or pharmacoeconomic or pharmaco‐economic$).ti,ab.
39. exp economics, hospital/
40. economics, medical/
41. economics, nursing/
42. economics, pharmaceutical/
43. (expenditure$ not energy).ti,ab.
44. (value adj1 money).ti,ab.
45. budget$.ti,ab.
46. (cba or cea or cua).ti,ab.
47. exp "fees and charges"/
48. (fee or fees or charge$ or preference$).tw.
49. (fiscal or funding or financial or finance).tw.
50. exp Health Care Costs/
51. (cost$ adj1 (util$ or effective$ or efficac$ or benefit$ or consequence$ or analy$ or minimi$ or saving$ or breakdown or lowering or estimate$ or variable$ or allocation or control or illness or sharing or life or lives or affordabl$ or instrument$ or technolog$ or day$ or fee or fees or charge or charges)).ti,ab.
52. ((value or values or valuation) adj3 (money or monetary or life or lives or costs or cost$)).ti,ab.
53. (unit cost or unit‐cost or unit‐costs or unit costs or drug cost or drug costs or hospital costs or health‐care costs or health care cost or medical cost or medical costs).ti,ab.
54. cost$.ti,ab.
55. exp decision support techniques/
56. markov$.ti,ab. or markov chains/
57. monte carlo.ti,ab. or monte carlo method/
58. (decision adj2 (tree$ or analy$ or model$)).ti,ab. or decision tree/
59. (survival adj3 analys$).ti,ab.
60. "deductibles and coinsurance"/
61. exp Health expenditures/
62. uncertain$.ti,ab. or uncertainty/
63. (quality adj3 life).ti,ab. or quality of life/
64. (value adj3 life).ti,ab. or value of life/
65. utilit$.tw.
66. valu$.tw.
67. exp hospitalization/
68. (qoly or qolys or hrqol or qaly or qalys or qale or qales or qald or qtime or daly or haly or hale or hqol or h‐qol or hr‐qol or hye or hyes).tw.
69. or/22–68
70. 4 and 12 and 21 and 69
71. limit 70 to yr="2009 ‐ Current"

### 2.3 Study selection

Articles were screened for eligibility based on criteria listed in Table S1. Titles and abstracts of all articles were reviewed for eligibility and only accepted if these criteria were met. Four reviewers (RH, DF, DS and ML) independently evaluated the full text of potentially eligible articles to determine whether to include or exclude. A lack of consensus over eligibility was resolved between the four reviewers. If doubts remained about study suitability (e.g. abstracts lacking peer review), they were excluded.

The integrity of each study was assessed according to a checklist developed by the ISPOR Consolidated Health Economic Evaluations Reporting Standards Task Force Report [15]. This underpinned development of a quality rating for each study, thus allowing rigorous evaluation of the robustness of the data generated. Quality assessment was performed by one reviewer, checked by a second reviewer and any disagreement resolved by third/fourth reviewers. Quality ratings were assigned in five categories (Table S2).

The Study Selection Workflow is outlined in Fig. S1. Our initial database search and other electronic searches (ASCO, ISPOR) identified 18 723 records. MEDLINE and Embase results were further searched based on health economic filters, as there is a paucity of these represented in the identified records. A total of 18 614 records were excluded and the remaining 110 records imported into reference management software, where duplicate records (*n* = 36) were removed. A total of 74 articles were screened for eligibility. After full text examination, 10 articles were either reviews or systematic reviews, which were retained for reference, whereas seven articles did not mention the terms life‐years gained (LYG), quality‐adjusted life‐years (QALY), or incremental cost‐effectiveness ratio (ICER). Seven other articles did not include cost‐effectiveness analysis (CEA), cost benefit analysis or cost utility analysis. On further examination, 12 were abstracts without enough detailed information, and one was an intervention without deployment of a PDT. In total, 37 eligible studies remained which involved economic evaluation of PDT for guiding therapeutic intervention in NSCLC.

### 2.4 Data extraction

We extracted empirical and methodological data and imported these data into Microsoft excel. Extracted features included: author, year, country of study, NSCLC stage/advanced/not described, therapy, biomarker utilised, LYG, QALY, the current ICER (cost per LYG) and/or ICER (cost per QALY), willingness‐to‐pay (WTP) cost‐effectiveness threshold (CET) and net monetary benefit (NMB) statistic (calculated based on LYG or QALY, costs and WTP). We also extracted author, PM cost, PDT cost (and calculated the PDT : PM cost ratio), perspective (healthcare payer, health insurance or hospital), modelling approach, time horizon (duration of therapy), discounting applied, one‐way sensitivity analysis (OWSA), probabilistic sensitivity analysis (PSA) and the trial upon which the economic evaluation was based. While most studies only listed one scenario of PDT intervention compared with standard of care (or another PDT), some studies listed as many as 10 different scenarios, where the scenario involved variation in the PDT, therapy or country. If there were insufficient data (e.g. abstract reports from conferences), we emailed the original authors for further details.

### 2.5 Data synthesis

Data capture and quality analysis for each study of cost‐effectiveness were represented in the data extraction and as a narrative summary. Modelling techniques used in the different studies were compared and their robustness analysed.

### 2.6 Sub‐analysis

Net monetary benefit was calculated in each instance where a PM‐guided by a PDT was compared with the same PM drug administered to all patients without PDT guidance.

### 2.7 Mathematical formulae employed

In cases where more than one therapy and test combination were modelled, the reported ICER might not be compared to the base case, e.g. best supportive care (BSC) or LYG and QALY reported, but no corresponding ICER calculated. In these instances, we calculated the ICER based on reported costings and QALY for the PDT using the following formula:

$$\text{ICER}=\frac{\Delta \text{Costs}}{\Delta \text{QALYs}\;\text{or}\;\Delta \text{LYGs}}.$$

For the studies that compared PDT‐guided therapy with unselected PDT therapy for all‐comers and with chemotherapy, we conducted a sub‐analysis using the NMB (a summary statistic that represents an intervention’s value in monetary terms) with the formula:

$$\text{NMB}=\left(\text{QALY}\;\text{or}\;\text{LYG}\times \text{WTP}\right)‐\text{Cost}.$$

The WTP CET employed for each scenario corresponded to that reported in the study; if more than one or a range of WTP CET were described, we conservatively chose the lowest. Additionally, if no WTP CET was disclosed, then the WTP CET from the same country in another captured study was employed, or 1× gross domestic product (GDP) per capita of that country was used.

## 3 Results

The 37 studies were reported from Asia, Australia, Europe and North America, all of which were at least upper middle‐income countries. Publications spanned the period 2009–2019. Where a negative ICER was reported or calculated, it was always due to negative costs, not negative LYG or QALY. The reader should refer to the NMB statistic before drawing conclusions, as a negative value here will always determine that the intervention was not cost‐effective.

### 3.1 Health outcomes for each precision medicine/precision diagnostic testing combination

#### 3.1.1 TKI treatment guided by *EGFR* status versus TKI treatment for all patients

Four of five ICER were negative (Table 2a), generated from increased QALY and less costly therapy; one of the ICER was positive [16, 17, 18, 19, 20]. Negative ICER can be equated with an intervention that can either be cost‐effective or not, which may lead to confusion. The simpler Net Monetary Benefit was calculated for each scenario; here a negative NMB indicates a non‐cost‐effective strategy. All four erlotinib studies produced positive NMB. The one gefitinib study evaluated revealed an NMB equal to an increase of Singapore dollars (S$)5800 per patient in value when the *EGFR* test is employed to guide gefitinib therapy, as opposed to an unselected approach (Table 2).

**Table 2:** Study characteristics and outcomes of TKI treatment guided by *EGFR* status versus TKI treatment for all patients. Negative ICER can infer favourable or unfavourable treatment options depending on where it lies on the cost‐effectiveness plane. Negative NMB implies intervention is not cost‐effective compared with standard of care. €, euros; NR, not reported; US$, US dollars.

Author	Year	Country	NSCLC stage	Therapy	Biomarker (methodology)	Change in LYG	Change in QALY	ICER (LYG)	ICER (QALY)	WTP CET^a^	NMB
Borget *et al*. [16]	2012	France	IIIB/IV	Erlotinib	*EGFR* DNA sequencing	0.150	0.081	−€38,767^b^	−€71,790^b^	€38,000^c^	€8,893
de Lima Lopes *et al*. [17]	2012	Singapore	Advanced	Gefitinib	*EGFR* ARMS PCR DxS *EGFR*29	NR	0.040	NR	−S$75,000^b^	S$75,000^c^	S$5,800
Hornberger *et al*. [18]	2015	USA	IIIB/IV	Erlotinib	*EGFR* Veristrat®	0.091	0.050	−US$1473^b^	−US$2680^b^	US$72,346	US$3,751
Lim *et al*. [19]	2016	South Korea	IIIB/IV	Erlotinib	*EGFR* DNA sequencing	NR	0.075	NR	−US$8733^b^	US$14,691^c^	US$1,749
Nelson, Stenehjemi, and Akerley. [20]	2013	USA	Advanced	Erlotinib	*EGFR* Veristrat®	0.108	0.090	US$75,926^b^	US$91,111	US$125,000	US$3,050

^a^ WTP CET is particular to each country.

^b^ ICER not reported and calculated from reported costs and LYG and/or QALY.

^c^ WTP CET not reported and the WTP CET from the same country was employed or 1× GDP per capita of that country.

#### 3.1.2 Immunotherapy treatment guided by PD‐L1 positivity versus immunotherapy treatment for all patients

Incremental cost‐effectiveness ratios generated by the PD‐L1 testing strategy were described as dominated (i.e. yielded worse health outcomes and were more costly) when compared with the no testing strategy when pembrolizumab treatment was considered. However, the corresponding incremental QALY and costs reported increased health benefits and were less expensive, respectively [21]. Nivolumab therapy accompanied by PD‐L1 testing generated ICER well below Swiss francs (CHF**)**100,000 WTP CET (Table 2b) [22].

**Fig. 1:**
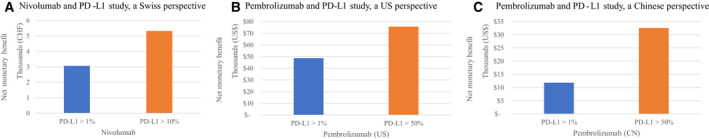
Sub‐analysis of health economic impact of PM. CN, China; PD‐L1 ≥ 1%, ≥ 10% and ≥ 50% figures refer to tumour proportion score; US$, US dollars. (A) Nivolumab and PD‐L1 study, a Swiss perspective. (B) Pembrolizumab and PD‐L1 study, a US perspective. (C) Pembrolizumab and PD‐L1 study, a Chinese perspective.

Sub‐analysis of the nivolumab study indicated an NMB of CHF86 per patient at PD‐L1 ≥ 1% and an NMB of CHF2,779 per patient at PD‐L1 ≥ 10% where the PD‐L1 test guides nivolumab therapy when compared with an unselected approach (Fig. 1A). Analysis of the pembrolizumab study revealed an NMB of US$32,604 per patient at PD‐L1 ≥ 1% and a NMB of US$56,889 per patient at PD‐L1 ≥ 50% in the USA (Fig. 1B). For China, the results indicated an NMB of US$27,039 per patient at PD‐L1 ≥ 1% and an NMB of US$52,120 per patient at PD‐L1 ≥ 50% (Fig. 1C).

In summary, PM treatments (erlotinib, gefitinib or immunotherapy) guided by a precision diagnostic test (*EGFR* or PD‐L1) increased the clinical and monetary value of PM for both the patient and healthcare payer when compared with an unselected treatment approach for ‘all’ patients (Table 3).

**Table 3:** Study characteristics and outcomes of immunotherapy guided by PD‐L1 positivity versus immunotherapy for all patients. Negative ICER can infer favourable or unfavourable treatment options depending on where it lies on the cost‐effectiveness plane. A dominated strategy is one which is clinically inferior and more expensive. CN, China; NR, not reported; US$, US dollars.

Author	Year	Country	NSCLC stage	Therapy	Biomarker (methodology)	Change in LYG	Change in QALY	ICER (LYG)	ICER (QALY)	WTP CET^a^	NMB
Matter‐Walstra *et al*. [22]	2016	Switzerland	NR	Nivolumab	PD‐L1 ≥ 1%, IHC PD‐L1 ≥ 10%, IHC IHC ‐ 22C3 pharmDx	(1%) 0.153^b^ (10%) 0.118^b^	0.100 0.090	CHF45,366^c^ CHF31,229^c^	CHF65 774 CHF37 860	CHF100,000	CHF86 CHF2779
Wan *et al*. [21]	2019	China	Advanced	Pembrolizumab	US: PD‐L1 ≥ 1% US: PD‐L1 ≥ 50% CN: PD‐L1 ≥ 1% CN: PD‐L1 ≥ 50% IHC ‐ 22C3 pharmDx	NR NR NR NR	US (1%) 0.270^b^ US (50%) 0.140^b^ CN (1%) 0.160^b^ CN (50%) 0.050^b^	NR NR NR NR	Dominated Dominated Dominated Dominated	US: US$100,000 CN: US$27 351	US$32 604 US$56 889 US$27 039 US$52 120

^a^ WTP CET is particular to each country.

^b^ Percentages in brackets refers to PD‐L1 expression cut‐off.

^c^ ICER not reported and calculated from reported costs and LYG and/or QALY.

#### 3.1.3 TKI treatment guided by *EGFR* or *ALK* status versus chemotherapy treatment

Of the 14 studies (20 scenarios) evaluated, 13 scenarios generated ICER which breached their respective WTP CET (Table 2c); this is reflected in their corresponding negative NMB values (Table 4).

**Table 4:** Study characteristics and outcomes of TKI treatment guided by *EGFR* or *ALK* status versus chemotherapy treatment. Negative NMB implies intervention is not cost‐effective compared to standard of care. A dominated strategy is one which is clinically inferior and more expensive. £, British pounds; ¥, Japanese Yen; €, euros; CAD$, Canadian dollars; CN, China; *EGFR*‐T790M, *EGFR* gatekeeper mutation; FISH, fluorescence *in situ* hybridization; NR, not reported; PAP, patient assistance programme; US$, US dollars

Author	Year	Country	NSCLC stage	Therapy	Biomarker (methodology)	Change in LYG	Change in QALY	ICER (LYG)	ICER (QALY)	WTP CET^a^	NMB
Arrieta *et al*. [23]	2016	Mexico	Advanced	Gefitinib	*EGFR* ARMS PCR DxS *EGFR*29	0.086	NR	US$55 349	NR	US$10,929^b^	−US$46,820^c^
Limwattananon *et al*. [24]	2018	Thailand	IIIB/IV	Gefitinib Erlotinib Afatinib	*EGFR* Test NR	0.111 0.123 0.191	0.148 0.193 0.208	US$82,964^d^ US$72,106^d^ US$43,221^d^	Dominated US$45,967 US$196,662	US$4,500	−US$8,543 −US$8,001 −US$10,872
Narita *et al*. [25]	2015	Japan	IIIB/IV	Gefitinib	*EGFR* ARMS PCR DxS *EGFR*29	NR	0.113	NR	¥3,380,00	¥5,000,000	¥225,000
Zhu *et al*. [26]	2013	China	Advanced	Gefitinib^e^ Gefitinib (PAP)^e^	*EGFR* ARMS PCR DxS *EGFR*29	0.750	0.740	US$34,867^d^ US$9,571^d^	US$57,066 US$15,665	US$18,951	−US$12,126 US$6,845
Handorf *et al*. [27]	2012	USA	IV	Erlotinib	*EGFR* FISH	NR	0.059	NR	US$110,658	US$100,000	−US$573
Schremser *et al*. [28]	2015	Germany	IV	Erlotinib	*EGFR* DNA sequencing of exons 18–21	NR	0.013	NR	€15,577	€70,500	€716
You *et al*. [29]	2019	China	Advanced	Afatinib	*EGFR* Therascreen *EGFR* 29	NR	0.150	NR	US$33,749	$26,508	US$1,093
Bertranou *et al*. [30]	2018	UK	Advanced	Osimertinib	*EGFR*‐T790M cobas mutation test	NR	1.541	NR	£41,705	£50,000	€12,768
Ezeife *et al*. [31]	2018	Canada	Advanced	Osimertinib	*EGFR*‐T790M plasma or tissue testing	1.040	0.790	CAD$169,610^d^	CAD$223,133	CAD$50,000	−CAD$136,894
Guan *et al*. [32]	2019	China	IIIB/IV	Osimertinib	*EGFR*‐T790M cobas mutation test	1.064	0.846	US$19,708^d^	US$24,976	US$30,000	US$5,081
Wu *et al*. [33]	2017	China	Advanced	Osimertinib	US: *EGFR*‐T790M CN: *EGFR*‐T790M cobas mutation test	0.877 0.877	0.704 0.642	US$178,072^d^ US$22,294^d^	US$222,030 US$30,472	US,‐,$100,000 CN,‐,$23,815	−US$85,769 −US$4,623
Wu *et al*. [34]	2019	China	Advanced	Osimertinib	US: *EGFR*‐T790M CN: *EGFR*‐T790M cobas mutation test	1.100 1.100	0.851 0.757	US$242,344^d^ US$28,596^d^	US$312,903 US$41,512	US,‐,US$150,000 CN,‐,US$30,000	−US$138,928 −US$8,746
Djalaov *et al*. [35]	2014	Canada	IV	Crizotinib	*ALK* IHC then FISH	0.640	0.379	CAD$148,011	CAD$250,632	CAD$100,000^b^	−CAD$57,143
Li, Lai, and Wu. [36]	2019	China	IIIB/IV	Alectinib Ceritinib	*ALK* ‐ FISH	0.890 0.810	1.000 1.090	US$69,922 US$18,722	US$62,231 US$13,905	US$28,410	−US$33,821 US$15,802

^a^ WTP CET is particular to each country.

^b^ WTP CET not reported and the WTP CET from the same country was employed or 1× GDP per capita of that country.

^c^ NMB calculated with LYG rather than QALY.

^d^ ICER not reported and calculated from reported costs and LYG and/or QALY.

^e^ 10‐year scenario.

#### 3.1.4 Immunotherapy treatment guided by PD‐L1 positivity versus chemotherapy treatment

Table 5 shows six scenarios which generated ICER below their WTP CET (Table 5), which correlated with positive NMB values; the remaining two scenarios had ICER which breached their WTP CET.

**Table 5:** Study characteristics and outcomes of immunotherapy guided by PD‐L1 positivity versus chemotherapy. Negative NMB implies intervention is not cost‐effective compared with standard of care. HK$, Hong Kong dollars; US$, US dollars

Author	Year	Country	NSCLC stage	Therapy	Biomarker (methodology)	Change in LYG	Change in QALY	ICER (LYG)	ICER (QALY)	WTP CET^a^	NMB
Aguiar *et al*. [37]	2017	USA	Advanced	Pembrolizumab	PD‐L1 ≥ 1% PD‐L1 ≥ 50% 22C3 pharm Dx IHC	0.690 0.809	0.350 0.409	US$49,007 US$41,187	US$98,421 US$80,735	US$100,000	US$981 US$7,579
Bhadhuri *et al*. [38]	2019	Switzerland	IV	Pembrolizumab	PD‐L1 ≥ 50% 22C3 pharm Dx IHC	1.700	1.340	CHF45,531	CHF57,402	CHF100,000	CHF56,940
Huang *et al*. [39]	2017	USA	IV	Pembrolizumab	PD‐L1 ≥ 50% 22C3 pharm Dx IHC	1.300	1.050	US$78,344	US$97,621	US$100,000	US$2,561
Loong *et al*. [40]	2019	Hong Kong	Advanced	Pembrolizumab	PD‐L1 ≥ 50% 22C3 pharm Dx IHC	0.360	0.280	HK$697,462	HK$865,189	HK$1,017,819	HK$35,912
She *et al*. [41]	2019	USA	Advanced	Pembrolizumab	PD‐L1 ≥ 1% PD‐L1 ≥ 20% PD‐L1 ≥ 50% 22C3 pharm Dx IHC	0.690 0.820 1.130	0.390 0.460 0.630	US$101,764 US$90,966 US$76,390	US$179,530 US$160,626 US$136,229	US$150,000	−US$12,387 −US$5,562 US$8,290

^a^ WTP CET is particular to each country.

#### 3.1.5 Treatment guided by genetic status using different testing scenarios

Twelve scenarios breached their WTP CET, two scenarios were dominated (clinically inferior and more expensive than the standard of care), and the remaining 11 scenarios were within their CET (Table 2e). The NMB indicated 13 scenarios that were not cost‐effective, 10 scenarios that were cost‐effective, and two scenarios which were marginal at zero (Table 6).

**Table 6:** Study characteristics and outcomes of treatment guided by genetic status using different testing scenarios. A dominated strategy is one which is clinically inferior and more expensive. Negative NMB implies intervention is not cost‐effective compared with standard of care. £, British pounds; €, euro; AUD$, Australian dollars; CAD$, Canadian dollars; *EGFR*‐TKI, *EGFR* TKI; FISH, fluorescence *in situ* hybridization; *HER2*, human epidermal growth factor receptor 2; HRM, high‐resolution melt; KRAS, kirsten rat sarcoma viral oncogene homolog; MTS, multiplex targeted sequencing; NR, not reported; PAP, patient assistance programme; qRT‐PCR, quantitative reverse transcription PCR; WAVE‐HS, sequencing methodology

Author	Year	Country	NSCLC stage	Therapy	Biomarker (methodology)	Change in LYG	Change in QALY	ICER (LYG)	ICER (QALY)	WTP CET^a^	NMB
Carlson *et al*. [50]	2009	USA	IIIB/IV	Erlotinib	*EGFR* – FISH IHC	0.120 0.080	0.060 0.040	US$76,742^b^ US$78,367	US$153,483^b^ US$162,018	$150,000	−US$209 −US$274
Westwood *et al*. [51]	2014	UK	IIIB/IV	*EGFR*‐TKI	*EGFR* ^c^					£30,000	
					1. Sanger seq. and fragment length analysis/PCR of −ve samples	NR	−0.007	NR	−£33,347		−£436
					2. HRM analysis	NR	−0.007	NR	−£30,143		−£421
					3. Sanger seq. or Therascreen PCR kit for samples with insufficient tumour cells	NR	−0.001	NR	−£45,629		−£70
					4. Therascreen PCR kit	NR	−0.001	NR	−£24,977		−£56
					5. Sanger seq. or Roche cobas for samples with insufficient tumour cells	NR	0.000	NR	Dominated		−£18
					6. Direct sequencing or WAVE‐HS	NR	0.000	NR	Dominated		0
					7. Direct sequencing of exons 19–21	NR	0.000	NR	£615,549		0
					8. Roche cobas	NR	0.001	NR	£19,501		£15
					9. Fragment length analysis combined with pyrosequencing	NR	0.001	NR	£79,807		−£32
					10. Single‐strand conformation analysis	NR	0.008	NR	£31,080		−£24
Lieberthal *et al*. [52]	2013	USA	Advanced	Targeted therapy	*EGFR* and *ALK* various	0.020	NR NR	US$154,512	NR	NR	−US$606e
Doble *et al*. [53]	2017	Australia	Advanced	Targeted therapy	Driver mutations other than *EGFR* or *ALK* MTS	0.008	0.009	AUD$485,199	AUD$489,338	AUD$100,000	−AUD$3403
Romanus *et al*. [54]	2015	USA	IV	Erlotinib or crizotinib	*EGFR*, *ALK* and other Overexpression assay, followed by FISH, and IHC	0.04	0.03	US$102,000	US$136,000	US$155,000	US$568
Loubiére *et al*. [55]	2018	France	Advanced	Gefitinib, erlotinib or crizotinib	*EGFR*, *ALK*, BRAF, HER2 and KRAS IHC, FISH and sequencing	0.197 0.162	NR NR	€13,320 €7,444	NR	€38,000	€4,884^d^ €4,962^d^
Roth *et al*. [56]	2014	USA	I/II	Chemotherapy	14‐Gene Risk Score Assay	0.150	0.080	US$11,952	US$23,154	US$50,000	US$2,191
Steuten *et al*. [57]	2019	USA	IIIB/IV	Targeted therapy	*EGFR*, *ALK*, BRAF, RET, ROS1, HER2 and MET multigene panel sequencing	0.060	NR	US$148,478	NR	US$100,000	−US$2,813^d^
Lu *et al*. [58]	2018	China	Advanced	Crizotinib (PAP)	*ALK*‐ multiplex PCR *ALK*‐ NGS panel	0.056 0.056	0.040 0.040	US$10,304b US$9,839b	US$14,384 US$13,740	$32,000	US$703 US$729
Lu *et al*. [59]	2016	China	IIIB/IV	Crizotinib (PAP)	*ALK*‐ Ventana (D5F3) CDx *ALK*‐ IHC plus FISH *ALK* – qRT‐PCR	0.051 0.050 0.048	0.029 0.028 0.027	US$9,667b US$9,580b US$13,979b	US$16,820 US$16,850 US$24,424	$32,000	US$435 US$417 US$193
Medical Advisory Secretariat[60]	2010	Canada	IIIB/IV	Gefitinib or erlotinib	*EGFR* PCR sequencing	0.054	0.031	CAD$46,021	CAD$81,017	CAD$50,000	−CAD$684

^a^ WTP CET is particular to each country.

^b^ The ICERs were not provided and were calculated from reported costs and QALYs.

^c^ Cost‐effectiveness of each test methodology compared with direct sequencing of all *EGFR* exon 18–21 mutations.

^d^ NMB calculated with LYG rather than QALY.

### 3.2 Analyses for each PM‐PDT cost evaluated

The annual cost of each study’s PM was identified and this was employed as a denominator to assess the fraction of test cost relative to precision therapy (Tables S3a–S3e). The proportion of PDT cost to therapy cost ranged from 0.03% [US$32 immunohistochemistry (IHC) test to detect *ALK* mutations to guide crizotinib therapy at US$86,966 in China] to 4.24% [US$550 amplification refractory mutation system (ARMS) PCR test to detect *EGFR* to guide gefitinib therapy at US$12,893 in Mexico]. IHC tests were consistently the least expensive, while costs increased with the complexity of the test employed.

### 3.3 Decision model for each PM‐PDT combination evaluated

The modelling approaches principally followed the Markov process (22 of the 37 articles, see Tables S3a–S3e). Five articles had a partition survival modelling (PSM) perspective, and four articles solely employed a decision analytic method. The remaining studies included a state‐transition model, a microsimulation with a state‐transition model, a microsimulation alone, a discrete event simulation, a decision Monte Carlo and an article which did not report its modelling approach.

### 3.4 Sensitivity analyses for each PM‐PDT combination evaluated

#### 3.4.1 TKI treatment guided by *EGFR* status versus TKI treatment for all patients

The one‐way sensitivity analyses (OWSA) and probabilistic sensitivity analyses (PSA) conducted indicated in four of five cases that PDT employed to guide therapy were the dominant strategy in France, Singapore, South Korea and USA, demonstrating clinical benefit and cost‐effectiveness for the patient (Table S3a) [16, 17, 18, 19, 20].

#### 3.4.2 Immunotherapy treatment guided by PD‐L1 positivity versus immunotherapy treatment for all patients

For immunotherapy guided by PD‐L1, the OWSA indicated that PDT deployment or patient’s health status had the greatest effect on the ICER, and the PSA determined that PD‐L1 testing increased cost‐effectiveness of the therapy in China, Switzerland and USA (Table S3b) [21, 22].

#### 3.4.3 TKI treatment guided by *EGFR* or *ALK* status versus chemotherapy treatment

##### Afatinib, erlotinib and gefitinib treatment guided by *EGFR* status

Reviewing the OWSA results, it was evident that both increasing mutation prevalence of *EGFR* and the health status of the patient had the greatest impact on the ICER. The PSA determined in four of seven studies that in China (with patient access programmes), Germany and Japan, *EGFR*‐guided therapy was cost‐effective, whereas the Mexican, Thai and US studies were not (Table S3c) [23, 24, 25, 26, 27, 28, 29].

##### Osimertinib treatment guided by *EGFR*‐T790M status

Overall, the OWSA results showed that the patient’s health status and the cost of osimertinib had the greatest effect on the ICER, whereas the PSA was inconclusive, with China and the UK studies cost‐effective, but Canadian, Chinese and USA studies not cost‐effective (Table S3c) [30, 31, 32, 33, 34].

##### Alectinib, ceritinib and crizotinib treatment guided by *ALK* status

The OWSA demonstrated that cost of therapy and patient’s health status influenced the ICER the most. Where a PSA was conducted in the Chinese study, it was likely that ceritinib was cost‐effective, whereas alectinib was not (Table S3c) [35, 36].

#### 3.4.4 Immunotherapy treatment guided by PD‐L1 positivity versus chemotherapy treatment

The OWSA performed showed that OS had a major impact on the ICER in four of five studies; in the four cases where PSA was conducted, the Swiss study was likely to be cost‐effective, Hong Kong and USA studies were inconclusive, and the USA study was not cost‐effective (Table S3d) [37, 38, 39, 40, 41].

#### 3.4.5 Treatment guided by genetic status using different testing scenarios

Although the OWSA in Canada, China and USA revealed that several ICER values were most sensitive to OS, PFS and drug costs, this was not true for all studies, with certain ICER values in Australia and France more impacted by high‐risk patients, inpatient care or costs alone. Most of the PSA performed suggested that these were not cost‐effective strategies, although the 14‐gene assay and *ALK* testing were cost‐effective (Table S3e).

## 4 Discussion

To our knowledge, there is no systematic review of economic evaluations of NSCLC which has focused specifically on PDT‐guided PM. In our analysis, we identified 64 CEA scenarios, evaluated within 37 studies, which satisfy our criteria, to determine ‘What is the cost‐effectiveness of PDT for guiding therapy in non‐small‐cell lung cancer?’ Thirty‐four (53%) of these scenarios were deemed cost‐effective. However, only 11 of the 64 scenarios followed the correct analysis format to assess whether a PDT adds value to a PM approach. That is, only these 11 scenarios compared PM and PDT with PM administered to the patient cohort without prior use of the test to select patients. Of these, seven scenarios (63.6%) agreed with our hypothesis of PDT‐guided treatment conferring measurable increased benefit. Four scenarios presented conflicting results of data from Wan *et al*.; [21] we believe that the authors may have mislabelled these studies as dominated (clinically inferior and more expensive) rather than dominant (less costly and better health outcomes), which corresponds to the incremental costs and QALY indicated in their results. The data that we have presented for these seven positive studies, and our conclusions, are supported by the authors of a recent systematic review of economic evaluation which only focussed on IO drugs. This study found that in NSCLC, molecular testing to help guide IO interventions provides more clinical benefit than the pharmaceutical agent alone [42].

Overall, the LYG or QALY gained for *EGFR*‐directed therapy were greater in the Asian studies than in North American or European populations, which is to be expected, as the prevalence of *EGFR* mutations is greater in Asia. In 59%(24 of 41 cases), the *EGFR‐*guided therapy failed cost‐effectiveness criteria regardless of test type; this is also true for *ALK* testing in 38% (five of 13) of cases, and in 14% (two of 14) of PD‐L1 testing (all IHC‐based testing).

A number of the testing scenarios involving next‐generation sequencing (NGS) have difficultly capturing more than one actionable mutation with standard CEA, as current Markov or state transition models aggregate patient data into distinct health groups [e.g. progression‐free survival (PFS), progressive disease (PD) and death], neglecting heterogeneity amongst the patient cohort, and PSM is incapable of returning to PFS from a PD state, where in some cases there is a distinct possibility of a ‘cure’. Dynamic simulation modelling such as discrete event simulation (DES) has recently been suggested as a model which can track individual patient pathways, incorporating results, testing and consequential therapies [43].

Our analyses strongly suggest that health economic evaluation should be performed routinely from the start of and alongside clinical trials. This is particularly true for precision oncology, where therapeutic costs are high and improved patient outcomes achieved through application of a relatively inexpensive PDT would be beneficial, both from a clinical and a health economic viewpoint. Previously, we have demonstrated a paucity of CEA studies for PM‐guided care in colorectal cancer; that same dearth of application of CEA is evident for NSCLC, with only seven of 37 studies (18.9%) adequately designed to analyse the cost‐effectiveness and value of PDT [14].

### 4.1 Strengths and weaknesses

The principal strength of this systematic review is that we employ the NMB summary statistic rather than the ICER to assess cost effectiveness. NMB incorporates both costs and QALY at a WTP threshold particular to that country, allowing cost‐effectiveness to be easily captured, thus generating more robust data. Secondly, we demonstrate that while PM is a driver of costs, PDT are a driver of value. PDT, at a fraction of the cost of a precision therapy, add value beyond the therapy, by selecting patients who will accrue greater health benefits and reducing costs by excluding patients who will not benefit from a particular PM approach.

Weaknesses of the data presented in this systematic review are that the majority of studies published are inappropriately structured to best assess effective PDT deployment in PM, which may reflect the lack of involvement of health economists and diagnostic stakeholders in setting the PM agenda.

Secondly, the generalisability of the results of this study is difficult to ascertain, as WTP CET vary not only between but also within countries where such studies are performed. WHO proposes that it is reasonable to spend income to achieve a QALY that is equivalent to the GDP per capita of a country, a recommendation followed in the UK by the National Institute for Health and Clinical Excellence with its £30,000 WTP CET, but this is adapted for end‐of‐life disease such as metastatic NSCLC at £50,000, and modified again for small patient subgroups, a hallmark of PM, with values of £75,000 upwards [44, 45]. Such high WTP CET could have a significant impact on the costs of a country’s healthcare system, if only the value of an intervention is considered. It would be advisable also to conduct a Budget Impact Analysis which would more robustly assess the intervention’s affordability [47].

Thirdly, all these CEA are based on randomised controlled trials (RCT) which involve highly selected patient populations. For PM RCT, the small patient populations and more complex clinical pathways may increase uncertainty in CEA modelling results. Adding CEA of real‐world data as an adjunct to RCT data would improve confidence in a treatment’s effectiveness [48].

Fourthly, the CEA do not capture capital costs (testing equipment), personnel and their training, and reporting tool costs.

Fifthly, patient waiting times between test and therapy and the impact of first and potentially further surgical biopsies are not reported, two important aspects of PDT deployment. The turnaround times from sequential single gene testing to NGS is important in advanced NSCLC, where appropriate speed of test turnaround may be crucial to a patient’s survival (and likely QALY impact). These are not modelled in the studies described. Liquid biopsies also add to the speed of tumour profiling, with the additional bonus of sampling being relatively painless to the patient [49].

## 5 Conclusion

Over half of the scenarios analysed presented ICER below the WTP CET, suggesting a potential publication bias which can only be addressed by increased diligence and transparency in the health economics/precision oncology evaluation. Only seven of 37 CEA studies performed to assess the benefit of PM approaches in NSCLC care were appropriately designed to assess the value of combining PDT with PM, highlighting the need for greater emphasis on precise health economic analysis to inform value‐based patient care. Despite this, employing molecular tests to guide NSCLC therapy appears to be cost‐effective in the majority of cases. Thus, cost‐effective deployment of PDT can add substantial value to the PM approach well in excess of the cost of the test itself and should inform a more robust approach for future PM delivery for NSCLC patients.

## Conflict of interest

ML reports honoraria received from Pfizer, EMD Serono, Roche and Carnall Farrar for presentations unrelated to this work. ML also reports an unrestricted educational grant from Pfizer for activity unrelated to this work. None of the other authors have any conflicts of interest to declare.

## Author contributions

RH, PK, DF, DS and ML were involved in the conception and design of the study. RH, DF, DS and ML created the search strategy and performed the article inclusion. RH performed the data extraction. RH, DF, DS and ML performed the quality assessment. All authors contributed to data interpretation, critically revised the article, and approved the final version.

## Supporting information

**Fig. S1**. PRISMA flow diagram
**Table S1**. Screening criteria and study design for systematic review.
**Table S2**. CHEERS criteria and quality rating.
**Table S3a**. Methodological characteristics and quality rating of TKI treatment guided by EGFR status versus TKI treatment for all patients.
**Table S3b.** Methodological characteristics and quality rating of immunotherapy guided by PD‐L1 positivity versus immunotherapy for all patients.
**Table S3c.** Methodological characteristics and quality rating of TKI treatment guided by EGFR or ALK status versus chemotherapy.
**Table S3d.** Methodological characteristics and quality rating of assessment of immunotherapy guided by PD‐L1 positivity versus chemotherapy.
**Table S3e.** Methodological characteristics and quality rating of treatment guided by genetic status using different testing scenarios.
