# Supplementary material for: Cost‐effectiveness of precision diagnostic testing for precision medicine approaches against non‐small‐cell lung cancer: A systematic review
Source: Mol Oncol. 2021 Jul 19;15(10):2672–87. doi: 10.1002/1878-0261.13038 (PMC8486593; doi:10.1002/1878-0261.13038)
Supplement: Supplementary file 1 — Fig. S1. PRISMA flow diagram Table S1. Screening criteria and study design for systematic review. Table S2. CHEERS criteria and quality rating. Table S3a. Methodological characteristics and quality rating of TKI treatment guided by EGFR status versus TKI treatment for all patients. Table S3b. Methodological characteristics and quality rating of immunotherapy guided by PD‐L1 positivity versus immunotherapy for all patients. Table S3c. Methodological characteristics and quality rating of TKI treatment guided by EGFR or ALK status versus chemotherapy. Table S3d. Methodological characteristics and quality rating of assessment of immunotherapy guided by PD‐L1 positivity versus chemotherapy. Table S3e. Methodological characteristics and quality rating of treatment guided by genetic status using different testing scenarios. [file MOL2-15-2672-s001.docx]

**Supplemental Data - Cost-effectiveness of precision diagnostic testing for precision medicine approaches against non-small-cell lung cancer: a systematic review**

**Figure S.1.** PRISMA flow diagram

**
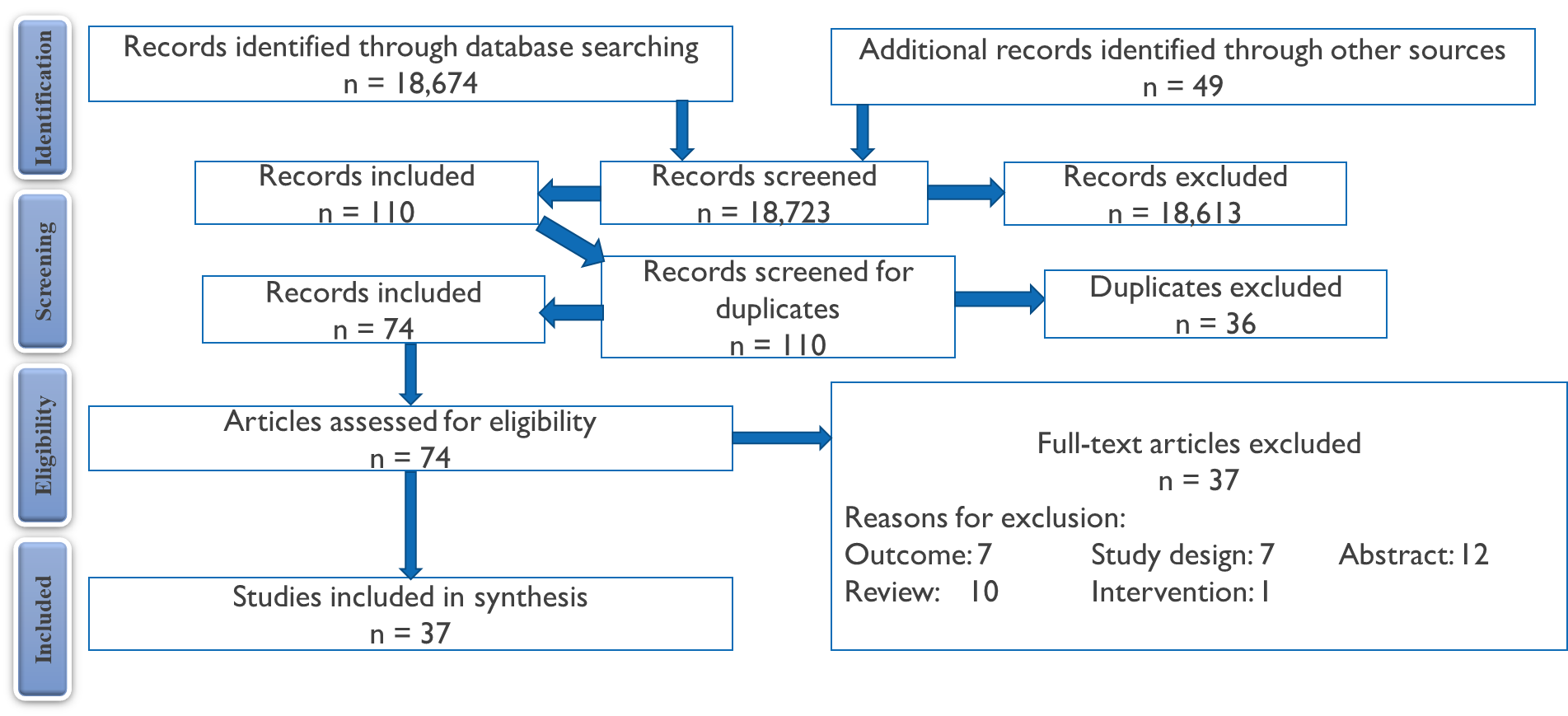
**

**Table S.1.** Screening Criteria and Study Design for Systematic Review

1. *Patients*: Diagnosed with NSCLC, not limited by age, gender, staging, or type of treatment intervention.
2. *Intervention*: PDTs including: Single or multi-gene tests (Cobas, Therascreen, High Resolution Melting Assay (HRMA), Sanger sequencing, pyrosequencing, NGS, multigene assays, mutational analysis); gene expression profiling; Fluorescence in situ hybridization (FISH); protein-based tests [immunohistochemistry (IHC)]. All other tests were excluded.
3. *Comparator*: no PDT.
4. *Outcomes*: The health economic indicator incremental cost-effectiveness ratio (ICER) was investigated, as it relates to cost per quality-adjusted life year (QALY) and cost per life year gained (LYG).
5. *Study design*: Screening for economic analyses based on models (which draw data from trials, resource use and health utility in a disaggregated form) or trials (which prospectively include all the required data). These included CEA, cost-benefit analysis (CBA), cost-minimization analysis (CMA) and cost-utility analysis (CUA). Budget-impact, reviews, letters and editorials were excluded from the systematic review, but were retained for reference.

**Table S.2.** CHEERS criteria and Quality Rating

| CHEERS criteria | Quality Rating | Interpretation |
| --- | --- | --- |
| 23 to 24 | ✔✔✔✔✔ | Excellent |
| 21 to 22 | ✔✔✔✔ | Good |
| 19 to 20 | ✔✔✔ | Medium |
| 17 to 18 | ✔✔ | Low |
| 16 or less | ✔ | Poor |

**Table S.3a.** Methodological characteristics and quality rating of TKI treatment guided by EGFR status versus TKI treatment for all patients

| Author | Annual cost of therapy | Cost of test | Test:Therapy ratio | Perspective | Modelling approach | Time horizon | Discount | OWSA | PSA | Clinical Trial | Quality rating |
| --- | --- | --- | --- | --- | --- | --- | --- | --- | --- | --- | --- |
| Borget et al.[16] | € 26,477 | € 130 | 0.49% | French 3rd  party payer | Markov | 2.5 years | 3% | Yes | Yes | ERMETRIC | ✓✓✓✓✓ |
| de Lima Lopes et al.[17] | S$34,900 | S$380 | 1.09% | NR | Decision analytic | NR | NR | Yes | No | IPASS | ✓✓✓✓ |
| Hornberger et al.[18] | US$80,245 | US$3,260 | 4.06% | US payer | NR | 3 years | 3% | Yes | Yes | PROSE | ✓✓✓✓ |
| Lim et al.[19] | US$25,726 | US$104 | 0.40% | Korean health- care payer | Markov | 5 years | 3% | Yes | No | NR | ✓✓✓✓✓ |
| Nelson, Stenehjemi,  and Akerley.[20] | US$164,328 | US$2,860 | 1.74% | US healthcare payer | Markov | NR | NR | Yes | Yes | PROSE | ✓✓✓✓ |

**ERMETIC** - Evaluation of EGFR mutation status for EGFR-TKI administration in NSCLC; **IPASS** - Open label, randomised, parallel group, multicentre, phase III study to assess efficacy, safety & tolerability of Gefitinib (IRESSA™) versus Carboplatin/Paclitaxel DC as 1st-line treatment in selected patients with stage IIIB / IV NSCLC in Asia; **PROSE** - Randomized proteomic stratified phase III study of second-line Erlotinib versus single-agent chemotherapy in patients with inoperable NSCLC.

€ - euro; NR – not reported; OWSA – one-way sensitivity analysis; PSA – probabilistic sensitivity analysis; S$ - Singapore dollars; US – United States; US$ - US dollars.

**Table S.3b.** Methodological characteristics and quality rating of immunotherapy guided by PD-L1 positivity versus immunotherapy for all patients

| Author | Annual cost of therapy | Cost of test | Test:Therapy ratio | Perspective | Modelling approach | Time horizon | Discount | OWSA | PSA | Clinical Trial | Quality rating |
| --- | --- | --- | --- | --- | --- | --- | --- | --- | --- | --- | --- |
| Matter-Walstra et al.[22] | CHF87,290 | CHF136 | 0.16% | Swiss healthcare | Markov | Lifetime | NR | Yes | Yes | CHECKMATE-057 | ✓✓✓✓ |
| Wan et al.[21] | US: US$169,815  CN: US$92,508 | US: US$60  CN: US$49 | US: 0.04%  CN: 0.05% | US and China public payers | Markov | Lifetime | 3% | Yes | Yes | KEYNOTE-189 | ✓✓✓✓✓ |

**CHECKMATE 057** - An open-label randomized phase III trial of BMS-936558 (Nivolumab) versus Docetaxel in previously treated metastatic Non-squamous NSCLC; **KEYNOTE-189** - A Randomized, Double-Blind, Phase III Study of Platinum + Pemetrexed Chemotherapy With or Without Pembrolizumab (MK-3475) in First Line Metastatic Non-squamous NSCLC Subjects.

CHF – Swiss Francs; CN – China; NR – not reported; OWSA – one-way sensitivity analysis; PSA – probabilistic sensitivity analysis; US – United States; US$ - US dollars.

**Table S.3c.** Methodological characteristics and quality rating of TKI treatment guided by EGFR or ALK status versus chemotherapy

| Author | Annual cost of therapy | Cost of test | Test:Therapy ratio | Perspective | Modelling approach | Time horizon | Discount | OWSA | PSA | Clinical Trial | Quality rating |
| --- | --- | --- | --- | --- | --- | --- | --- | --- | --- | --- | --- |
| Arrieta et al.[23] | US$12,893 | US$550 | 4.24% | Mexico and Latin America | Discrete event  simulation | 2 years | 5% | Yes | Yes | IPASS | ✓✓✓ |
| Limwattanon et al.[24] | US$20,089 US$20,454 US$25,933 | US$225 | 1.12% 1.10% 0.87% | Thailand societal and healthcare | Markov | 5 years | 3% | Yes | Yes | 10 RCTs pooled | ✓✓✓✓ |
| Narita et al.[25] | ¥2,383,695 | ¥21,000 | 0.88% | Japanese healthcare payer | Markov | 5 years | 2% | Yes | Yes | IPASS | ✓✓✓ |
| Zhu et al.[26] | US$28,416 | US$508 | 1.79% | Chinese health- care system | Markov | 10 years | 3% | Yes | Yes | INFORM; C-TONG 0804 | ✓✓✓✓✓ |
| Handorf et al.[27] | US$52,791 | US$243 | 0.46% | US payer | Decision analytic | NR | NR | Yes | Yes | BR.21 and IPASS | ✓✓✓✓ |
| Schremser et [28]al. | € 22,390 | € 323 | 1.44% | German SHI | State transition  model | 10 years | 3% | Yes | Yes | EURTAC | ✓✓✓✓✓ |
| You et al.[29] | US$17,773 | US$316 | 1.78% | Chinese health- care system | Markov | 5 years | 5% | Yes | Yes | LUX-Lung 6 | ✓✓✓✓ |
| Bertranou et al.[30] | £70,250 | £1,351 | 1.92% | UK NHS | PSM | Lifetime | 3.50% | No | Yes | AURA, AURA2,  and IMPRESS | ✓✓✓✓✓ |
| Ezeife et al.[31] | CAD$107,276 | CAD$300 | 0.28% | Canadian publically  funded health care system | Markov | 10 years | 1.5% | Yes | Yes | FLAURA | ✓✓✓ |
| Guan et al.[32] | US$20,478 | US$282 | 1.38% | Chinese health- care system | Markov | 10 years | 3% | Yes | Yes | AURA AURA2 | ✓✓✓✓ |
| Wu et al.[33] | US$186,278 US$94,600 | US$966 US$441 | 0.52% 0.47% | US payer and  Chinese health-care system | Decision tree and Markov | 10 years | US - 3% CN - 5% | Yes | Yes | AURA3 | ✓✓✓✓✓ |
| Wu et al.[34] | US$207,462 US$94,599 | US$966 US$441 | 0.47% 0.47% | US payer and  Chinese health-care system | Decision tree and Markov | 10 years | US - 3% CN - 5% | Yes | Yes | AURA3 FLAURA | ✓✓✓✓✓ |
| Djalaov et al.[35] | CAD$121,333 | IHC: CAD$40 FISH: CAD$388 | 0.03% 0.32% | Canadian Public health (Ontario) | Decision analytic  and Markov | Lifetime | 5% | Yes | No | PF-02341066 | ✓✓✓✓✓ |
| Li, Lai, and Wu.[36] | US$95,550 US$31,905 | NR |  | Chinese health- care system | Markov | 10 years | 5% | Yes | Yes | ASCEND 4 & 8,  PROFILE 1014, PARAMOUNT | ✓✓✓✓✓ |

**ASCEND 4** - Phase III multicenter, randomized study of oral LDK378 versus standard chemotherapy in previously untreated adult patients with ALK rearranged (ALK-positive), Stage IIIB or IV, Non-squamous NSCLC; **ASCEND 8** - Randomized phase 1 study of Ceritinib, 450 mg or 600 mg, taken with a low-fat meal versus 750 mg in fasted state in patients with ALK-rearranged metastatic NSCLC; **AURA** - Phase I/II open-label, multicentre study to assess the safety, tolerability, pharmacokinetics and anti-tumour activity of ascending doses of AZD9291 in patients with advanced NSCLC who have progressed following prior therapy with an EGFR-TKI Agent; **AURA2** - Phase II, open label, single-arm study to assess safety and efficacy of AZD9291 in patients with locally advanced/metastatic NSCLC whose disease has progressed with previous EGFR TKI and whose tumours are EGFR and T790M mutation positive; **AURA3** - Phase III, open label, randomized study of AZD9291 versus Platinum-based doublet chemotherapy for patients with locally advanced or metastatic NSCLC whose disease has progressed with previous EGFR-TKI therapy and whose tumours harbour a T790M mutation within the EGFR gene; **BR.21** - International, phase 3, randomized, double-blind, placebo-controlled trial of erlotinib after the failure of first-line or second-line chemotherapy for NSCLC; **EURTAC** - Phase III, multicenter, open-label, randomized trial of Tarceva® vs chemotherapy in patients with advanced NSCLC with mutations in the TK domain of the EGFR; **FLAURA** - Phase III, double-blind, randomised study to assess the efficacy and safety of AZD9291 versus a standard of care EGFR-TKI as first-line treatment in patients with EGFR mutation positive, locally advanced or metastatic NSCLC. **INFORM;C-TONG 0804** - Phase III, randomized, placebo-controlled, parallel-group study of gefitinib versus placebo as maintenance therapy in patients with locally advanced or metastatic NSCLC; **IPASS** - Open label, randomised, parallel group, multicentre, phase III study to assess efficacy, safety & tolerability of Gefitinib (IRESSA™) versus Carboplatin/Paclitaxel DC as 1st-line treatment in selected patients with stage IIIB / IV NSCLC In Asia; **IMPRESS** - A Phase III randomised, double blind, placebo controlled, parallel, multicentre study to assess the efficacy and safety of continuing IRESSA 250 mg in addition to chemotherapy versus chemotherapy alone in patients who have EGFR mutation positive locally advanced or metastatic NSCLC and have progressed on first line IRESSA; **LUX-Lung 6** - Randomized, open-label, phase iii study of BIBW 2992 versus chemotherapy as first-line treatment for patients with stage IIIB or IV adenocarcinoma of the lung harbouring an EGFR activating mutation; **PF-02341066** - Phase 1b open-label study of the safety and clinical activity of Crizotinib in tumors with genetic events involving the ALK gene locus; **PARAMOUNT** - Phase 3, double-blind, placebo-controlled study of maintenance Pemetrexed plus best supportive care versus best supportive care immediately following induction treatment with Pemetrexed + Cisplatin for advanced NSCLC; **PROFILE 1014** - Phase 3, randomized, open-label study of the efficacy and safety of Crizotinib versus Pemetrexed/Cisplatin or Pemetrexed/Carboplatin in previously untreated patients with non-squamous carcinoma of the Lung harboring a translocation or inversion event involving the ALK gene locus.

ALK - Anaplastic Lymphoma Kinase; CAD$ - Canadian dollars; CN – China; €- euros; EGFR – epidermal growth factor receptor; EGFR-TKI – EGFR tyrosine kinase inhibitor; EGFR-T790M – EGFR gatekeeper mutation; FISH - fluorescence *in situ* hybridization; IHC – immunohistochemistry; ¥ - Japanese Yen; NHS – national health insurance; NR – not reported; OWSA – one-way sensitivity analysis; PSA – probabilistic sensitivity analysis; PSM – partition survival modelling; RCT -randomised controlled trial; SHI – social health insurance; US – United States; US$ - US dollars.

**Table S.3d.** Methodological characteristics and quality rating of assessment of immunotherapy guided by PD-L1 positivity versus chemotherapy

| Author | Annual cost of therapy | Cost of test | Test:Therapy ratio | Perspective | Modelling approach | Time horizon | Discount | OWSA | PSA | Clinical Trial | Quality rating |
| --- | --- | --- | --- | --- | --- | --- | --- | --- | --- | --- | --- |
| Aguiar et al.[37] | NR | NR |  | US Medicare system | Decision analytic | 5 years | 10% or 20% | Yes | No | KEYNOTE 010 | ✓✓✓✓ |
| Bhadhuri et al.[38] | CHF90,445 | NR |  | Swiss healthcare payer | PSM | 20 years | 3% | Yes | Yes | KEYNOTE 024 | ✓✓✓✓✓ |
| Huang et al.[39] | US$151,875 | NR |  | US third-party  public healthcare payer | PSM | 20 years | 3% | Yes | Yes | KEYNOTE 024 | ✓✓✓ |
| Loong et al.[40] | HK$1,016,219 | HK1,700 | 0.17% | Hong Kong Hospital Authority | PSM | 10 years | 3% | Yes | Yes | KEYNOTE 024 | ✓✓✓✓✓ |
| She et al.[41] | US$339,643 | US$315 | 0.09% | US payer's | Markov | 20 years | 3% | Yes | Yes | KEYNOTE 042 | ✓✓✓ |

**KEYNOTE 010** - Phase II/III randomized trial of two doses of MK-3475 (SCH900475) versus Docetaxel in previously treated subjects with NSCLC; **KEYNOTE 024** - Randomized open-label phase III trial of MK-3475 versus Platinum based chemotherapy in 1l subjects with PD-L1 strong metastatic NSCLC; **KEYNOTE 042** - Randomized, open label, phase III study of overall survival comparing Pembrolizumab (MK-3475) versus platinum based chemotherapy in treatment naïve subjects with PD-L1 positive advanced or metastatic NSCLC.

CHF – Swiss Francs; CN – China; HK$ - Hong Kong dollars; NR – not reported; OWSA – one-way sensitivity analysis; PSA – probabilistic sensitivity analysis; PSM – partition survival modelling; US – United States; US$ - US dollars.**Table S.3e.** Methodological characteristics and quality rating of treatment guided by genetic status using different testing scenarios

| Author | Annual cost of therapy | Cost of test | Test:Therapy ratio | Perspective | Modelling approach | Time horizon | Discount | OWSA | PSA | Clinical Trial | Quality rating |
| --- | --- | --- | --- | --- | --- | --- | --- | --- | --- | --- | --- |
| Carlson et al.[50] | US$35,234 | US$320 US$97 | 0.91% 0.28% | US societal | Decision analytic and PSM | 2 years | 3% | Yes | Yes | BR.21 | ✓✓✓✓ |
| Westwood et al.[51] | NR | £140  £150 £137   £155 £130  £148 £148 £140 £188  £140 |  | NHS | Decision tree and Markov | 1 year | 3.50% | No | Yes | NR | ✓✓✓✓ |
| Lieberthal et al.[52] | US$37,500 | US$470 | 1.25% | Managed care payer | Decision analytic | 1 year | NR | Yes | No | NR | ✓✓✓ |
| Doble et al.[53] | AUD$72,384 | AUD$1,000 | 1.38% | 3rd party Australian  healthcare payer | Decision tree and Markov | 10 years | 5% | Yes | No | Lung Cancer  Mutation Consortium | ✓✓✓✓ |
| Romanus et al.[54] | US$104,557 | EGFR IHC: US$201 ALK IHC: US$136 ALK FISH: US$489 | 0.19% 0.13% 0.47% | Societal | Microsimulation state-transition | 2 years | 3% | Yes | No | NR | ✓✓✓✓ |
| Loubiére et al.[55] | € 40,529 | EGFR: €181 KRAS: €213 ALK: €111 | 0.47% 0.53% 0.27% | Healthcare payer's | Microsimulation | NR | NR | Yes | Yes | PREDICT.amm | ✓ |
| Roth et al.[56] | US$4,296 | US$3,995 | 92.99% | Payer | Markov | Lifetime | 3% | Yes | Yes | JBR.10 | ✓✓✓✓✓ |
| Steuten et al.[57] | US$117,863 | US$1,948 | 1.65% | US payer | Decision monte carlo | Lifetime | 3% | Yes | Yes | Various | ✓✓✓ |
| Lu et al.[58] | US$86,966 | PCR: US$661 NGS: US$1,014 | 0.76% 1.17% | Chinese health- care system | Markov | 10 years | 5% | Yes | Yes | NR | ✓✓✓✓ |
| Lu et al.[59] | US$86,966 | Ventana: US$32 IHC+FISH: US$492 qRT-PCR: US$397 | 0.04% 0.57% 0.46% | Chinese health- care system | Markov | 10 years | 5% | Yes | Yes | PROFILE 1014 | ✓✓✓✓✓ |
| Medical Advisory Secretariat[60] | CAD$19,555 | CAD$500 | 2.56% | Ontario Ministry of  Health and Long-Term Care | Markov | Lifetime | 5% | No | Yes | IPASS BR.21 | ✓ |

**BR.21** - international, phase 3, randomized, double-blind, placebo-controlled trial of erlotinib after the failure of first-line or second-line chemotherapy for NSCLC; **IPASS** - open label, randomised, parallel group, multicentre, phase III study to assess efficacy, safety & tolerability of Gefitinib (IRESSA™) versus Carboplatin/Paclitaxel DC as 1st-line treatment in selected patients with stage IIIB / IV NSCLC in Asia; **JBR.10** - Phase III prospective randomized study of adjuvant chemotherapy with Vinorelbine and Cisplatin in completely resected NSCLC with companion tumour marker evaluation; **LCMC** - Lung Cancer Mutation Consortium established in 2008 as a multi-institutional program investigating the frequency of selected oncogenic drivers in lung ACA and using the results to treat the enrolled subjects with targeted therapies, either as part of standard clinical care or on investigational protocols; **PREDICT.amm** - Cross-validation study, KRAS, EGFR and ALK molecular status were assessed in 843 consecutive patients with previously untreated advanced NSCLC; **PROFILE 1014** - Phase 3, randomized, open-label study of the efficacy and safety of Crizotinib versus Pemetrexed/Cisplatin or Pemetrexed/Carboplatin in previously untreated patients with non-squamous carcinoma of the Lung harboring a translocation or inversion event involving the ALK gene locus.

AUD$ – Australian dollars; CAD$ – Canadian dollars; CET – cost effectiveness threshold; EQ-5D – European quality-of-life 5-dimension; LYG – life-years gained; NHS- National Health Service; NR – not reported; OWSA – one-way sensitivity analysis; PSA – probabilistic sensitivity analysis; PSM – partition survival modelling; QLQ-C30 - European Organization for the Research and Treatment of Cancer Quality of Life Questionnaire; SG – standard gamble; VAS – visual analogue scale; WTP – willingness-to-pay; US – United States
